# Prefrontal Cortex Responses to Social Video Stimuli in Young Children with and without Autism Spectrum Disorder

**DOI:** 10.3390/brainsci14050503

**Published:** 2024-05-16

**Authors:** Candida Barreto, Adrian Curtin, Yigit Topoglu, Jessica Day-Watkins, Brigid Garvin, Grant Foster, Zuhal Ormanoglu, Elisabeth Sheridan, James Connell, David Bennett, Karen Heffler, Hasan Ayaz

**Affiliations:** 1 School of Biomedical Engineering, Science, and Health Systems, Drexel University, Philadelphia, PA 19104, USA; 2 A.J. Drexel Autism Institute, Philadelphia, PA 19104, USA; 3 St. Christopher’s Hospital for Children, Philadelphia, PA 19134, USA; 4 School of Education, Drexel University, Philadelphia, PA 19104, USA; 5 Department of Psychiatry, College of Medicine, Drexel University, Philadelphia, PA 19129, USA; 6 Department of Psychological and Brain Sciences, College of Arts and Sciences, Drexel University, Philadelphia, PA 19104, USA; 7 Drexel Solutions Institute, Drexel University, Philadelphia, PA 19104, USA; 8 Center for Injury Research and Prevention, Children’s Hospital of Philadelphia, Philadelphia, PA 19104, USA

**Keywords:** autism spectrum disorder (ASD), functional near infrared spectroscopy (fNIRS), prefrontal cortex (PFC), medial PFC (mPFC), social neuroscience

## Abstract

Autism spectrum disorder (ASD) is a neurodevelopmental disorder affecting individuals worldwide and characterized by deficits in social interaction along with the presence of restricted interest and repetitive behaviors. Despite decades of behavioral research, little is known about the brain mechanisms that influence social behaviors among children with ASD. This, in part, is due to limitations of traditional imaging techniques specifically targeting pediatric populations. As a portable and scalable optical brain monitoring technology, functional near infrared spectroscopy (fNIRS) provides a measure of cerebral hemodynamics related to sensory, motor, or cognitive function. Here, we utilized fNIRS to investigate the prefrontal cortex (PFC) activity of young children with ASD and with typical development while they watched social and nonsocial video clips. The PFC activity of ASD children was significantly higher for social stimuli at medial PFC, which is implicated in social cognition/processing. Moreover, this activity was also consistently correlated with clinical measures, and higher activation of the same brain area only during social video viewing was associated with more ASD symptoms. This is the first study to implement a neuroergonomics approach to investigate cognitive load in response to realistic, complex, and dynamic audiovisual social stimuli for young children with and without autism. Our results further confirm that new generation of portable fNIRS neuroimaging can be used for ecologically valid measurements of the brain function of toddlers and preschool children with ASD.

## 1. Introduction

Autism Spectrum Disorder (ASD) is a pervasive neurodevelopmental disorder with global implications. According to the Diagnostic and Statistical Manual of Mental Disorders (DSM-5), ASD is characterized by a series of combined symptoms encoded into two main groups: (1) persistent deficits in social communication and social interaction across multiple contexts, and (2) restricted, repetitive patterns of behavior, interests, or activities [1]. The prevalence of ASD in children has been dramatically increasing worldwide [2]. In the USA, for instance, one out of every thirty-six children was diagnosed with ASD by age 8 years in 2020 against one out of sixty-eight in 2010 and one out of a hundred and fifty in 2000 [3,4]. Alongside genetic factors, these behavioral patterns are proposed to stem from deficits in neural factors underpinning deficiencies in both nonsocial and social cognition [5]. Nonetheless, pinpointing the exact causes of ASD remains challenging, partly because of the significant variability in clinical phenotype and the constraints of current technologies for assessing the neural mechanisms, particularly at earlier ages [6,7].

Difficulties with social interaction is one of the core symptoms of ASD. Children within the autism spectrum may have difficulty understanding social cues and norms, making it challenging to establish and maintain relationships with others [6]. They may also have difficulty with nonverbal communication such as making eye contact and using and interpreting facial expressions and body language. For instance, ASD children produced less emotional and more ambiguous facial expressions than typically developing (TD) children during social tasks such as interacting with an adult [8] and imitating facial productions [9]. ASD children also presented poorer performance than TD children on tasks involving emotion recognition of facial expressions [10,11,12,13]. Different from TD children, ASD children have also demonstrated preferences for nonsocial stimuli such as geometric patterns and objects rather than social stimuli such as human or animal faces [14,15,16]. It is noteworthy that the severity of social difficulties among children with ASD varies widely, with some ASD children developing successful social relationships while others may experience social isolation, exclusion, and difficulty making and keeping friends [17].

Recent advances in neuroimaging techniques have allowed the non-invasive investigation of the brain mechanisms underlying the social processing of ASD individuals. Several studies have applied non-invasive brain technologies such as Functional Magnetic Resonance Imaging (fMRI) and Electroencephalography (EEG) to investigate the brain function differences of ASD (see reviews [18,19,20]). Altered activation and connectivity patterns of regions related to the brain referred to as the social brain, including the prefrontal cortex, inferior frontal gyrus, and amygdala, have been observed in ASD individuals [21,22,23]. Cortical networks from EEG signals have presented different connectivity patterns in ASD compared to TD groups [24]. Most of these studies, however, were with adults, adolescents, and older children, and the experimental paradigms relied on tasks with non-dynamic stimuli such as images, pictures, or simple auditory stimuli. Therefore, more naturalistic studies emulating real-life situations with dynamic stimuli are needed to enhance the understanding of the neural correlates involved in social processing among toddlers and young children with ASD.

The limited number of naturalistic studies examining the social brain of ASD children might be explained by specific requirements of fMRI and EEG systems, including their high operational costs, noise, and movement restrictions that are challenging for toddlers and children. Alternatively, functional near infrared spectroscopy (fNIRS) is an emerging neuroimaging technology that is cost-effective, more resistant to movement artifacts, and accessible for measuring brain activity in real-world settings, consistent with a neuroergonomics approach [25,26,27,28,29]. As such, fNIRS has been applied to measure brain activity among both healthy and clinical populations of children [30,31,32,33]. There are also dedicated reviews of fNIRS studies among individuals with autism [34,35,36]. According to a 2019 review, at least thirty published studies applied fNIRS to measure ASD or high-risk children between 2006 and 2018 [34]. fNIRS has been used to measure brain activity in social contexts such as social perception among infants at-risk for ASD comparing social and nonsocial context stimuli, ASD children interacting with a human or a robot in a video, and live interaction between ASD children and their parents [31,37]. Also, fNIRS has been deployed to investigate the impact on social processing of infants in low-resources and at-risk of ASD and ADHD [38,39]. Although fNIRS offers promising avenues for investigating the social brain function within the ASD population, the limited number of naturalistic studies with toddlers and preschoolers underscores the need for further exploration.

This neuroergonomics study’s contributions are aimed to be threefold: (1) evaluating young children with ASD at first diagnosis age, (2) using complex dynamic and continuous stimuli, and (3) assessing localized prefrontal cortex activity for social cognition. Most fNIRS studies of social processing among children with ASD have focused on either infants or older children and adolescents, creating a critical need for more studies with ASD toddlers and preschoolers [40], which is the age when many children are first diagnosed. Moreover, previous studies utilized simple stimuli with short-duration clips or static photos that are more appropriate for infants, but not for toddlers and preschoolers. In this study, we implement a neuroergonomic approach to investigate the cognitive load in response to realistic, complex, and dynamic audiovisual social stimuli for toddlers, and preschoolers. We studied the brain hemodynamic responses of ASD and TD children while watching video clips of social and nonsocial contexts. Our aim was to evaluate prefrontal cortex (PFC) activity in response to naturalistic stimuli. The PFC, specifically the medial prefrontal cortex (mPFC), plays a crucial role in human social thinking and actions [41,42]. However, information about the association between this region and social cognition in children with autism is still underexplored [40]. We expected that ASD children would exhibit altered PFC activation patterns compared to TD children and that such differences may vary between social and nonsocial stimuli.

## 2. Materials and Methods

### 2.1. Participants

Twenty-eight children, twelve (nine male) ASD (3.89 y ± 1.26) and sixteen (six male) TD (2.88 y ± 0.63), participated. Regarding participant recruitment and selection procedures, we used a combination of approaches, such as advertising through online platforms, social media, and outreach to early childcare agencies and ASD community organizations. Potential participants’ parents and caregivers who contacted our research team were informed about the study and answered questions to verify their eligibility. Exclusion criteria for the ASD group included additional developmental disorders, severe medical conditions (e.g., history of seizures, traumatic brain injury, stroke), hearing/visual problems, and children on neuropsychiatric medications. For TD children, the exclusion criteria included having a family history of ASD and/or the presence of language or developmental delays. Once eligibility was confirmed, a mock headband was shipped to participants’ homes to help the ASD children get familiarized with the fNIRS headband. Eligible participants were invited for a remote session where a clinical psychologist with expertise in ASD assessment conducted a confirmatory evaluation of the children using the TELE-ASD-PEDS [43] and the Vineland Adaptive Behavior Scores, Third Edition (Vineland™-3) [44,45] to confirm ASD diagnosis. After the clinical assessments, participants were invited to the session in the lab with fNIRS data collection. Participants were monetarily compensated for their time. The Drexel University Institutional Review Board approved the study, and caregivers provided written consent for their and their child’s participation.

### 2.2. Experimental Setup

Participants were comfortably seated in front of a computer monitor on which the videos were displayed. Children viewed four video clips, two clips each for social and nonsocial conditions. We carefully chose all the video stimuli. For the social condition, one video with a male actor and another with a female actress were selected to counterbalance across our sample. The videos used for nonsocial conditions were gender neutral, as they included only inanimate general objects which are equally common for boys and girls (e.g., Rube Goldberg chain reaction machine). To keep participants’ engagement, we chose, for both social and nonsocial conditions, videos that presented a sequence of events. Each video lasted approximately two minutes; time markers were used to separate each block condition and were later used in the fNIRS analysis. Videos were presented in counter-balanced pseudo-randomized order. The whole experiment was video recorded by high-resolution cameras (Logitech HDPro Webcam C920, Logitech, Lausanne, Switzerland). The webcam video recordings of children’s faces were used to confirm that children were watching throughout the videos.

### 2.3. fNIRS Data Collection

**Figure 1:**
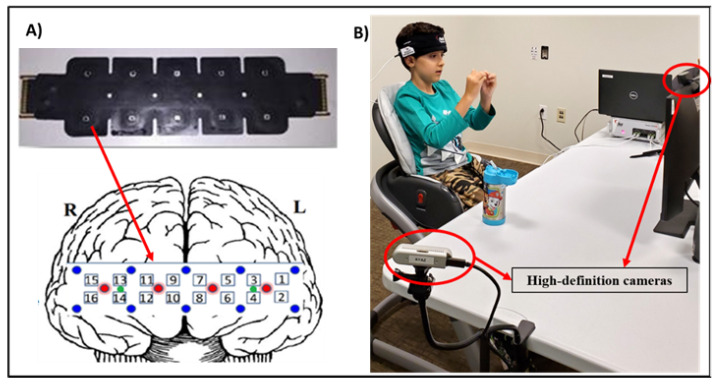
(**A**). fNIRS flat headband sensor and equivalent positions in the prefrontal cortex (left panel). Red and blue dots represent light emitters and detectors, respectively. Numbers (1–16) in between each pair represent the fNIRS optodes. (**B**). Data collection setup with a pilot participant in front of screen and camera (right panel).

Brain activity was monitored while the children viewed the videos. Prefrontal hemodynamic responses were measured using a continuous wave fNIRS Imager Model 2000S (fNIR Devices, LLC, Potomac, MD, USA) with wavelengths of 730 nm and 850 nm as well as an ambient channel with a sampling rate frequency of 10 Hz using COBI Studio Modern Software (v1.2) [46]. The prefrontal cortex activity was measured by placing an ultra-thin flat sensor pad on the participant’s forehead. The sensor pad measured 16 cortical measurement locations (optodes) with four sources and ten detectors at a 2.5 cm source–detector distance [47]. The sensors arrangement followed the 10–20 EEG system and covered the PFC bilaterally (see Figure 1A).

All participants went through brief head sensor acclamation training before data collection day. First, a mockup sensor (an elastic fabric headband) was shipped to participant’s home. Before the day of data collection, participants and parents practiced putting it on during an online meeting with a Board-Certified Behavior Analyst to ensure children can tolerate the sensor during the data collection.

### 2.4. fNIRS Processing and Statistical Analysis

For each participant, raw fNIRS light intensity data (16 optodes × 2 light wavelengths) were low pass filtered with a finite infinite response (FIR) filter order of 100 and cut-off of 0.1 Hz to attenuate high frequency noise and physiological artifacts [48]. Each participant’s data were checked for any potential saturated channels and contamination by using the additional ambient channel at each of the 16 optodes. Next, a coefficient of variation-based statistical filter, sliding-window motion artifact rejection (SMAR), was applied to the light intensity of each optode for rejecting motion artifacts, as we described previously [49]. Then, preprocessed light intensity time-series were converted into oxyhemoglobin changes (HbO) by applying the modified Beer–Lambert law. The hemodynamic response at each optode was averaged across time for each video block to provide a mean hemodynamic response at each optode. The final output of each optode was mean HbO which was used for statistical analyses.

A linear mixed model (LMM) analysis with repeated measures was applied to the HbO signal from each optode separately, with the variables group (ASD vs. TD) and condition (Social vs. Nonsocial) as fixed factors and age as a covariate. We used an LMM because it is particularly useful for examining brain hemodynamic changes over time as noted in this review of statistical analysis of fNIRS data [50]. LMMs incorporate random effects for individual participants while capturing variability between groups of participants [51,52]. Furthermore, LMM is flexible in handling missing data [52]. It does not require the same number of measurements across participants, which applies to fNIRS studies in which data may be missing due to technical issues or participant non-compliance. Finally, LMM has been frequently applied in fNIRS studies, including those assessing motor control [53] and cognitive processing [54,55].

False discovery rate (FDR) correction was applied to LMM results to correct for multiple testing familywise across the entire list of optodes (with *q* = 0.1) [56]. Bonferroni correction was applied during the post hoc analysis of individual contrast comparisons within an optode that survived the previous FDR correction [50].

### 2.5. Correlation between fNIRS Signals and Clinical Scores

In addition, we investigated the relationship of brain activity with clinical measures. Correlational analyses between HbO responses and clinical scores were performed for all participants and in each social and nonsocial condition. For each of the sixteen optodes, Spearman correlations were computed between HbO and both TELE-ASD-PEDS total Likert scores and Vineland™-3 clinical domains. Statistical analyses were performed using NCSS 2023. 

## 3. Results

### 3.1. Clinical Assessments Results

**Figure 2:**
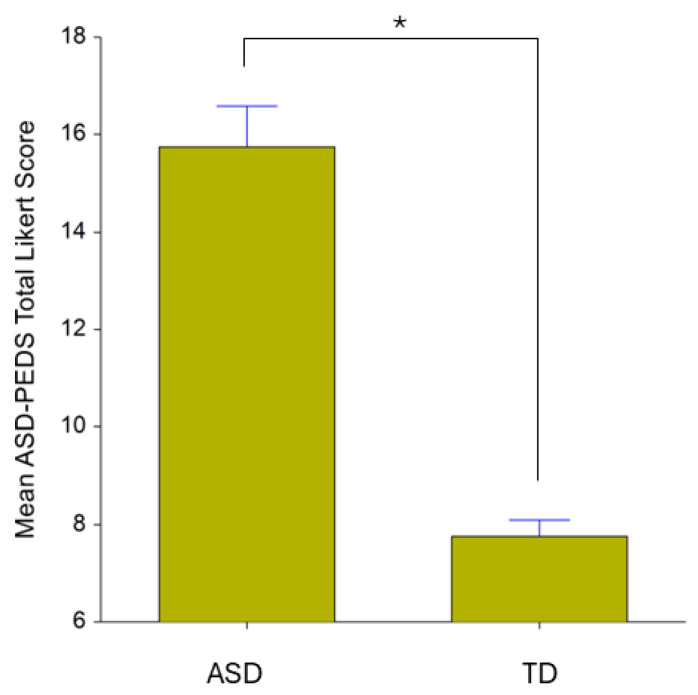
Mean TELE-ASD-PEDS Total Likert Scores. ASD = Autism Spectrum Disorder group, TD = typically developing group. * t (14.81) = 8.84, *p* < 0.001.

A comparison of ASD and TD children on the Vineland Adaptive Behavior Scales is presented in Table 1. Statistically significant differences in all domains and subdomains were found between ASD and TD groups. As expected, ASD group had lower mean scores than TD in all domains, indicating worse adaptive functioning. In addition, children with ASD had significantly higher scores than TD children on the TELE-ASD-PEDS (see Figure 2), further supporting the diagnoses of ASD among children in the ASD group.

### 3.2. fNIRS Results

**Figure 3:**
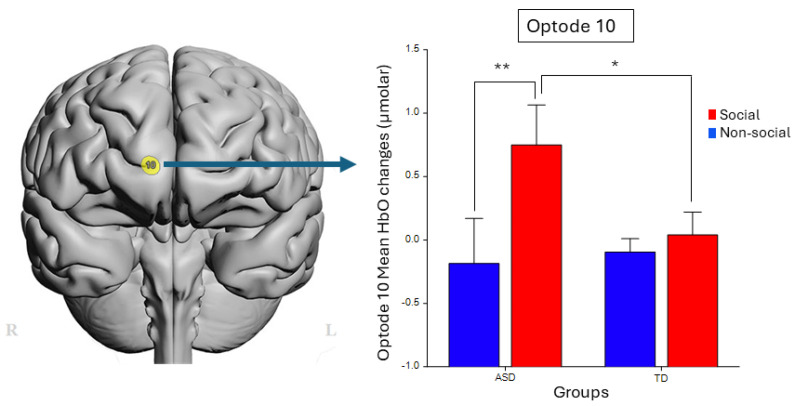
Projection of optode 10 on the brain surface image (**left**) and mean HbO results (**right**) by *group* (ASD × TD) and *condition* (Social × Nonsocial) shows significant interaction (F_1,69.6_ = 9.27, *p* = 0.003, η^2^ = 0.12), and with post hoc comparisons, significantly higher ASD-Social compared to ASD-Nonsocial (**, F_1,70.2_ = 19.61, *p* < 0.0001, η^2^ = 0.22) and significantly higher ASD-Social compared to TD-Social (*, F_1,39_ = 6.78, *p* < 0.03, η^2^ = 0.15).

Significant main effects of *condition* were found for HbO signals from optodes 2 (F_1,91_ = 12.09, *p* < 0.001, η^2^ = 0.12), 3 (F_1,88_ = 11.31, *p* = 0.001, η^2^ = 0.11), 5 (F_1,92_ = 8.42, *p* = 0.005, η^2^ = 0.08), 8 (F_1,74.1_ = 6.58, *p* = 0.012, η^2^ = 0.08), and 10 (F_1,70_ = 13.92, *p*-value < 0.001, η^2^ = 0.17), controlled for age and survived correction for multiple comparisons (FDR, q < 0.1). In all these optodes, the social *condition* resulted in higher activity compared to the non-social *condition*. More importantly, the interaction effect of group*condition was found to be significant only at optode 10 (F_1,69.6_ = 9.27, *p* = 0.003, η^2^ = 0.12). Post hoc comparison tests for optode 10 with Bonferroni correction showed that ASD children specifically presented higher HbO levels for the ASD social condition compared to the ASD nonsocial condition (**, F_1,70.2_ = 19.61, *p* < 0.0001, η^2^ = 0.22), while the TD group did not present statistically significant differences across social vs. nonsocial conditions. In addition, post hoc comparison of the ASD-Social condition was significantly higher compared to TD-Social (*, F_1,39_ = 6.78, *p* < 0.03, η^2^ = 0.15), as shown in Figure 3. No other optode had a significant response.

### 3.3. Correlation between fNIRS and Clinical Scores

First, fNIRS responses during social video conditions (Social HbO) showed positive correlations with TELE-ASD-PEDS scores only for optodes 10 (*r* = 0.37, *p* = 0.008) and 13 (*r* = 0.30, *p* = 0.044); the more ASD symptoms, the higher brain activation during social video viewing. For non-social condition HbO data, there was no significant correlation for any optode.

Next, correlations of fNIRS with standard scores from the Vineland™-3 were performed. Again, social HbO showed a significant correlation with the overall Adaptive Behavior Composite score and only for optode 10 (*r* = −0.031, *p* = 0.026) with poorer adaptive behavior associated with higher brain activation. There were no significant correlations for non-social videos.

Likewise, social HbO and Vineland™-3 domains showed significant correlation only for optode 10 for Socialization SS (*r* = −0.31, *p* < 0.030) and Communication SS (*r* = −0.33, *p* = 0.021) and for the relevant subdomain scales of Socialization Interpersonal Relations (*r* = −0.31, *p* < 0.030) and Communication Receptive (*r* = −0.30, *p* = 0.034).

The negative correlation between Social HbO and Vineland™-3 scores align with the correlations with TELE-ASD-PEDS and indicates that children with lower adaptive functioning (consistent with greater ASD symptoms) presented higher PFC activity during social videos. Again, there were no significant correlations for non-social HbO with the clinical measures, suggesting that the association between clinical scores and brain activity differences is specific to the social stimuli.

For Motor Skill SS, there was a significant relationship with HbO at optode 1 (*r* = 0.33, *p* < 0.025) for social conditions and no significant optodes for non-social conditions. For the Daily Living Skills SS domain, there were no significant correlations for social or non-social conditions. This is expected as these domains are not related to social and communication skills. None of the significant correlations survived after FDR for familywise optode multiple testing correction; however, consistent brain area (optode 10 for social cognition) and condition (only for social videos, and none for non-social video) suggest a confirmatory relationship to the main brain activity results.

## 4. Discussion

This neuroergonomic study aims to understand the neural correlates of social stimuli processing in ASD by evaluating young children (toddlers and preschoolers using dynamic stimuli and assessing prefrontal cortex activity with minimally intrusive wearable neuroimaging in ecologically valid settings. The brain activity of the young children with and without ASD was recorded as they watched social and non-social audiovisual stimuli, namely video clips. Our findings indicate that children with ASD exhibit distinct activation patterns for social and nonsocial stimuli. Children with ASD demonstrated higher HbO while watching social than nonsocial content, whereas no differences between viewing conditions were found for TD children. Additionally, significant negative correlations between adaptive functioning and children’s brain responses were found only for social videos and only for the same brain area (optode 10, measuring the medial PFC [mPFC]). These indicate that children whose Vineland™-3 clinical scores were lower (more severe symptoms) had higher brain hemodynamic responses to social videos. Moreover, higher activation of the same brain area (mPFC) during social video viewing was again associated with higher TELE-ASD-PEDS scores (i.e., more ASD symptoms).

Brain activity recorded during video watching also showed a significant interaction of group (*ASD* vs. *TD*) and condition (*Social* vs. *non-social*) in mPFC (optode 10, see Figure 3). These findings are consistent with prior research indicating that the mPFC plays an essential role in social cognition [57]. The mPFC activity has been associated with social stimuli from very early in life [40,58]. For example, the mPFC showed greater activation among 5-month-old infants while viewing a video with directed eye gazing than without directed gaze [58]; also, the mPFC was recruited when infants heard a voice calling their name but not when calling a stranger’s name [58].

Furthermore, the mPFC has been implicated as a relevant region in ASD-related fMRI studies. For instance, greater activation of mPFC was found in the ASD group when comparing a stimulus-oriented versus stimulus-independent attention measure [59]. Also, abnormal functional specialization within the mPFC was found among individuals with ASD when using multi-voxel analysis to compare brain activation of verbal versus spatial tasks [60]. Altered functional connectivity of the mPFC and other regions of the default mode network among individuals with ASD was also found in an fMRI study [61,62]. In ASD, general hyper-connectivity, specifically between the primary sensory cortex with paralimbic systems and the association cortex, has been reported [63]. Both social disability and repetitive behavior scores have been associated with hyper-connectivity patterns [63,64]. Also, ASD has been shown to have structural cortical differences, i.e., in a postmortem study, children with ASD presented significantly more neurons in the PFC than controls [65]. It is noteworthy that the mPFC is just one aspect of the broader neural basis of ASD, and more investigation is needed to build a deeper understanding of brain function differences among individuals with ASD.

Our results suggest that children with ASD demand more brain resources to process social cues than when in nonsocial situations (See Figure 3). Notably, the significant inverse relationship between HbO and adaptive functioning, particularly in communication skills, reinforces these findings (See Section 3.3). In the social videos, a person promotes interaction with children by talking and singing, perhaps creating greater cognitive challenges and load among individuals with ASD who may have some communication deficits. Likewise, the correlation between brain activity and Vineland™-3 socialization scores may elucidate the difficulties of ASD children in processing the actors’ social cues, such as facial expressions and gestures, during the social videos. In contrast, nonsocial videos did not contain human voices or social cues, possibly explaining the absence of a significant correlation between brain responses and clinical scores for that condition.

The pattern of differences in brain activity between social and nonsocial stimuli for the ASD group but not for the TD group has been reported in other studies [37,66,67,68]. For instance, in an fNIRS study that investigated brain hemodynamic responses of ASD and TD children while viewing videos with humans and robots, ASD children presented higher levels of HbO for the human condition. At the same time, no differences were found between the human and robot conditions in the TD group [37]. Also, brain activity measured by EEG while watching videos with social and nonsocial contexts was different for ASD and TD children [67]. Similarly, in an fMRI experiment, ASD children exhibited higher levels of PFC activity for pictures of human faces than pictures of houses. In contrast, the TD children had opposite patterns [68].

Furthermore, behavioral studies of preferences for social and nonsocial stimuli show that individuals with ASD have lower preferences for social than nonsocial stimuli, while TD peer preferences vary across studies [16,69,70]. For instance, when comparing preferences between social and nonsocial stimuli, TD children presented similar preferences, while the ASD group presented preferences for nonsocial stimuli [15]. Similarly, in a choose-a-movie paradigm that measures social seeking, adolescents with ASD preferred viewing objects to smiling faces, and, in contrast, TD adolescents showed no differences across stimuli conditions [66]. The higher PFC activity for social videos seen in our study may relate to the lower preference or engagement/experience of ASD children for social cues, therefore requiring higher cognitive demand to process it. TD children, on the other hand, may equally prefer social and nonsocial videos, leading to a similar level of PFC activity to process both stimuli.

The advantages of portable neuroimaging, such as fNIRS, have contributed to understanding the neural underpinnings of social processing in children as early as the first months of life [71]. The neural correlates of processing social stimuli have been shown, using fNIRS, to be related to psychosocial hazards (e.g., maternal education, maternal stress, socio-economic status, and the caregiving environment) [38,72,73]. For instance, higher brain hemodynamic responses to social stimuli as compared to nonsocial stimuli were found in infants (6 months) and toddlers (36 months) exposed to adversities, including extreme poverty, malnutrition, recurrent infections, and low maternal education [38]. In a longitudinal study comparing low and middle-income, the 6-month infants from both groups had similar functional connectivity patterns during a social cognition task; however, at 24 months, different connectivity patterns were observed, including evidence of greater global connectivity (less neural specialization) associated with environmental adversity [72]. Similarly, another fNIRS study of infants from the first postnatal days to the second year of life showed different activation patterns to social and nonsocial stimuli across different cohorts [73]. While the 9–24 months group demonstrated higher brain activity for social than nonsocial stimuli (both visual and auditory), younger infants from 0 to 2 months demonstrated higher activity for nonsocial auditory stimuli, which continued until 8 months [73].

Other fNIRS studies have investigated the brain responses to social stimuli of infants at elevated likelihood of atypical development, including those at risk for ASD and ADHD [39,74,75]. For instance, infants (4–6 months) at high risk for autism showed greater hemodynamic response to nonvocal stimuli and diminished response to visual stimuli in a short video paradigm in which visual and auditory stimuli are presented separately [74]. The same pattern of activation was found at 5 months in a sample at high risk of autism when applying a similar paradigm; while high-risk infants showed decreased activation to social stimuli in the right posterior temporal cortex, this activation was increased in infants at low risk of ASD [76]. Following the same direction, infants (4–6 months) with an elevated likelihood of ASD and/or ADHD showed atypical social brain responses to visual stimuli when compared to infants at typical development likelihood [39].

The divergence between our results of higher brain hemodynamic response to social videos in the ASD group and the reduced brain response to social stimuli reported in infant studies can be explained by several factors. Firstly, the differences in age range between our study (mean age ± 36 months) and infant studies should be considered given major brain developmental changes during this period. Secondly, we were interested in measuring the cognitive load of social processing. Therefore, we monitored children’s prefrontal cortex, which plays an important role in cognitive load. In contrast, the main differences in brain responses of the infants’ studies were found in lateral regions covered by the relative positions of the EEG 10–20 system such as T3–T4 [74,75,76]. Thirdly, our study used relatively long (2 min for each video), complex, and dynamic audio-visual stimuli trying to resemble a real-life situation. The infant studies used briefer videos that included only visual cues (human gestures) or auditory cues at a time, as it was more appropriate to their age range. However, these stimuli do not reflect the social complexity that older children encounter.

While this study provides insights into the neural responses of children with ASD to social and nonsocial stimuli, some limitations should be considered. Here, we described important albeit preliminary information about fNIRS measures of the PFC hemodynamic response and its relationship to activation patterns during complex social and non-social audiovisual stimuli processing. It is possible that there may be additional factors that contribute to the cognitive load and children may have recruited areas of the brain that were not being monitored in the current study. Examination of posterior brain regions such as the temporal–parietal junction (TPJ) and subcortical areas could provide additional information regarding young ASD children’s processing of social information in addition to the prefrontal cortex, or by combining fNIRS with EEG, as these measure complimentary aspects of brain activity [55]. This is an area for future research. Moreover, our participant recruitment was impacted by the COVID pandemic, limiting our sample size and composition. Future studies could investigate naturalistic social stimuli processing in ASD with larger participant pools and include additional multimodal wearable sensors. Finally, although our results have middle to large and large effect sizes, one should still interpret those with caution. Mixed-effects models with covariates effectively account for multiple sources of variation. Yet, due to their ability to discern among these varied factors, establishing a standardized effect size is challenging [77].

## 5. Conclusions

In conclusion, our study demonstrates that differential responses to social and nonsocial video stimuli are present in the brain of young children with ASD, and specifically in the mPFC, an area is involved with social cognition. The mPFC activity was also consistently correlated with clinical measures. Collectively, these findings contribute to the understanding of social cognition among young children with ASD and underscore the importance of investigating the neural mechanisms underlying naturalistic social stimuli processing. These findings could contribute to the development of potential early assessment approaches, interventions, and educational materials that effectively engage and support individuals with ASD. Follow up studies should continue to investigate the underlying mechanisms driving these response patterns. Future research with a multidisciplinary approach incorporating educational, clinical, and neuroscientific perspectives may contribute to the development of potential interventions that are tailored to the individual needs of children with ASD, promoting their social interaction, learning, and overall well-being.

## Figures and Tables

**Table 1:** Vineland Adaptive Behavior Scores, Third Edition (Vineland™-3).

	ASD (*n* = 12) Mean (SD)	TD (*n* = 16) Mean (SD)	T-Statistics (Two Sample)	DF	*p*-Value
Adaptive Behavior Composite	64.42 (6.14)	104.75 (16.41)	−9.03	20.17	<0.001
Domain Scores					
Communication SS	58.42 (15.39)	105.44 (11.82)	−8.81	20.02	<0.001
Daily Living Skills SS	72.92 (10.77)	101.31 (15.76)	−5.62	25.84	<0.001
Socialization SS	60.67 (10.35)	105.25 (15.51)	−9.11	25.73	<0.001
Motor Skills SS	81.00 (7.78)	105.44 (15.71)	−5.40	23.05	<0.001
Subdomain Score Summary					
Communication Receptive	7.25 (3.86)	15.63 (2.8)	−6.36	19.20	<0.001
Communication Expressive	4.17 (3.04)	16.38 (3.32)	−10.10	24.89	<0.001
Communication Written	12.50 (3.1)	16.25 (2.12)	−3.04	15.70	<0.001
DLS Personal	8.85 (3.78)	15.44 (3.48)	−4.51	22.78	<0.001
DLS Domestic	10.00 (2.21)	15.88 (2.64)	−5.03	13.70	<0.001
DLS Community	9.50 (2.88)	15.38 (1.6)	−5.49	14.51	<0.001
Socialization Interpersonal Relations	6.83 (2.76)	16.50 (4.03)	−9.88	24.75	<0.001
Socialization Play and Leisure	7.83 (1.59)	15.94 (2.72)	−9.89	24.75	<0.001
Socialization Coping Skills	8.73 (1.74)	15.46 (2.63)	−7.49	20.89	<0.001
Motor Skills Gross Motor	11.33 (2.77)	15.94 (3.55)	−3.85	25.93	<0.001
Motor Skills Fine Motor	12.08 (2.78)	16.50 (3.29)	−3.85	25.56	<0.001

*t*-test: Aspin–Welch Unequal-Variance *t*-Test, SS = Standard Score, DLS = Daily Living Skills Domain, ASD = Autism Spectrum Disorder group, TD = typically developing group.

## Data Availability

The original contributions presented in the study are included in the article/Supplementary Materials, further inquiries can be directed to the corresponding author.

## Supplementary Materials

The following supporting information can be downloaded at: https://www.mdpi.com/article/10.3390/brainsci14050503/s1. Table S1: Additional demographic information.
