# Supplementary material for: Prefrontal Cortex Responses to Social Video Stimuli in Young Children with and without Autism Spectrum Disorder
Source: Brain Sci. 2024 May 16;14(5):503. doi: 10.3390/brainsci14050503 (PMC11119834; doi:10.3390/brainsci14050503)
Supplement: Supplementary file 1 [file brainsci-14-00503-s001.zip › brainsci-2986724-supplementary.pdf]

Supplementary Material for

Prefrontal cortex responses to social video stimuli in young children with and without autism spectrum disorder

Candida Barreto[1], Adrian Curtin[1], Yigit Topoglu[1], Jessica Day-Watkins[2], Brigid Garvin[3], Grant Foster[1], Zuhal Ormanoglu[1], Elisabeth Sheridan[2], James Connell Jr.[4], David Bennett[5], Karen Heffler[5], and Hasan Ayaz[1,2,6-9, *]

Table S1. Additional demographic information of participants

**Demographics**	**ASD (n=12)**	**TD (n=16)**
Race<br>Afro American<br>Asian<br>White<br>Multiple	 2 (16.6%%)<br>2 (16.6%)<br>6 (50.0%)<br>2 (16.6%)	 3 (18.75%)<br>3 (18.75%)<br>6 (37.5%)<br>4 (25%)
Maternal age when the child was born.<br>(mean ± sd) in years	 30 ± 4.41	 32.9 ± 5.43
Pregnancy (weeks):<br>Baby's birth weight<br>Low<br>Normal<br>High	39.5 (2.393)<br><br>1 (8.33%)<br>9 (75%)<br>2 (16.66%)	38.81(1.680)<br><br>1 (6.25%)<br>15 (93.75%)
